# Comparative Phenotypic Characterization of Five CRISPR/Cas9-Generated Gene-Disrupted Mutants of Pseudorabies Virus JM Strain

**DOI:** 10.3390/vetsci13090867

**Published:** 2026-08-26

**Authors:** Longfei Han, Shanghui Wang, Zhaoxia Zhang, Changjiang Weng

**Affiliations:** 1 Heilongjiang Provincial Key Laboratory of Veterinary Immunology, Harbin 150069, China; 2 Division of Fundamental Immunology, National African Swine Fever Para-Reference Laboratory, State Key Laboratory of Animal Disease Control and Prevention, Harbin Veterinary Research Institute, Chinese Academy of Agricultural Sciences, Harbin 150069, China

**Keywords:** pseudorabies virus, virulence gene, CRISPR/Cas9, gene-disrupted mutant, UL21, UL24, neurovirulence, immune evasion

## Abstract

Pseudorabies virus (PRV) is a neurotropic alphaherpesvirus that causes severe neurological and reproductive disease in swine and can infect a broad range of non-porcine mammals. To compare the phenotypic consequences associated with disruption of different PRV genes within a uniform genetic background, UL21, UL24, UL50, UL56, and gG (US4) were individually disrupted in the PRV JM strain using CRISPR/Cas9. The resulting gene-disrupted viruses were characterized by multi-step growth analysis, plaque formation, and experimental infection in mice. The five viruses displayed distinct and non-parallel phenotypic profiles. PRV JM-ΔUL24, PRV JM-ΔUL50, PRV JM-ΔUL56, and PRV JM-ΔgG showed reduced peak extracellular viral titers, whereas PRV JM-ΔUL21 exhibited only a limited change in viral growth. In contrast, markedly smaller plaques were observed for PRV JM-ΔUL21 and PRV JM-ΔUL56, indicating that extracellular viral yield and plaque phenotype were not necessarily concordant. In mice, PRV JM-ΔUL21 and PRV JM-ΔUL24 showed delayed mortality and 40% survival, whereas PRV JM-ΔUL50 caused delayed but complete mortality and PRV JM-ΔUL56 and PRV JM-ΔgG retained survival patterns similar to parental PRV JM. Thus, attenuation in vivo was not directly predicted by impaired replication or plaque formation in vitro. Among the five gene-disrupted viruses examined, PRV JM-ΔUL21 and PRV JM-ΔUL24 showed the most pronounced attenuation-associated phenotypes in the mouse model, although substantial residual virulence remained. These comparative findings identify UL21 and UL24 as priority loci for further mechanistic evaluation and for assessment in future multi-gene attenuation strategies.

## Simple Summary

Pseudorabies virus (PRV) is an important swine pathogen that can also infect a broad range of non-porcine mammals. In this study, five PRV genes, UL21, UL24, UL50, UL56, and gG, were individually disrupted in the same PRV JM genetic background using CRISPR/Cas9, and the resulting viruses were comparatively characterized in vitro and in mice. The five gene-disrupted viruses exhibited distinct phenotypic profiles in viral replication, plaque formation, and survival in mice. Notably, changes in viral growth and plaque phenotype in cultured cells were not necessarily associated with attenuation in vivo. Among the viruses examined, PRV JM-ΔUL21 and PRV JM-ΔUL24 showed comparatively greater attenuation in mice, although substantial residual virulence remained. These findings demonstrate the value of evaluating candidate attenuation-associated loci across multiple phenotypic levels and provide comparative information for prioritizing UL21 and UL24 for further mechanistic investigation and future multi-gene attenuation studies.

## 1. Introduction

Pseudorabies (PR), also known as Aujeszky’s disease, is an acute febrile infectious disease caused by pseudorabies virus (PRV) and is characterized by reproductive disorders in sows and respiratory and neurological symptoms in piglets [1]. Pigs are the natural host of PRV infection and the main source of viral transmission. However, PRV can also cause pseudorabies in other livestock and various wild animals, with infections in non-porcine animals typically resulting in fatal outcomes.

The PRV genome is approximately 145 kb in size, with a high GC content of around 73% [2]. The PRV genome is primarily composed of two regions: the UL and US regions. Multiple virulence-related genes have been identified in PRV, including UL10, UL13, UL21, UL23, UL24, UL27, UL44, and UL56 in the UL region and US3, US7, and US8 in the US region [3]. Among these virulence-related genes, US7 (gI), US8 (gE), and UL23 (TK) have been extensively studied and are considered crucial virulence genes in the development of PRV gene-deletion vaccines. Although PRV with deletions in US7/US8/UL23 can protect pigs from wild-type PRV infection, it poses a significant threat to other susceptible animals, such as dogs, mink, and foxes [4]. These observations highlight the need to better understand the genetic determinants governing PRV virulence and attenuation and to develop more rational attenuation strategies.

The development of rationally attenuated PRV strains requires an understanding of how individual viral genes contribute to replication, spread, and pathogenicity. However, most virulence-associated genes have been investigated separately, often in different viral backgrounds or experimental systems, making their relative phenotypic contributions difficult to compare directly. UL21, UL24, UL50, UL56, and gG were therefore selected for comparative analysis because previous studies have implicated these genes in different aspects of PRV replication, virus–host interactions, and virulence [3,5,6,7,8]. Comparative analysis within the same PRV JM genetic background may help distinguish their relative contributions to viral fitness and virulence and identify candidate loci for subsequent multi-gene attenuation strategies.

CRISPR/Cas9 technology is a biotechnological tool for gene editing and modification aimed at precisely altering target genomes. As one of the third-generation gene editing technologies, CRISPR/Cas9 offers several advantages, such as low cost, high efficiency, and simplicity. The application of CRISPR/Cas9 in PRV editing was first carried out by Xu et al., and its applications in PRV include the construction of recombinant viruses, exploration of virulence genes for vaccine development, and the study of virus protein functions [9].

Therefore, in the present study, UL21, UL24, UL50, UL56, and gG were individually disrupted in the PRV JM background using CRISPR/Cas9 technology. The resulting viruses were comparatively evaluated in terms of replication kinetics, plaque formation, virulence, and tissue pathology. The primary objective was to compare the phenotypic effects associated with disruption of these five genes and to provide information for the rational selection of candidate loci for future multi-gene attenuation strategies.

## 2. Materials and Methods

### 2.1. Viruses, Cells, and Plasmids

PK-15 cells, 293T cells, and Vero cells were cultured in DMEM (Gibco, Grand Island, NY, USA) supplemented with 10% fetal bovine serum (FBS; Gibco, Grand Island, NY, USA) in an incubator containing 5% CO_2_ at 37 °C. 293T cells were used for co-transfection of PRV genomic DNA and pX330-sgRNA plasmids during recombinant virus construction. PK-15 cells were used for plaque purification, propagation of recombinant viruses, and plaque-size assays. Vero cells were used for TCID_50_ titration of virus-containing supernatants. The PRV JM parental strain was propagated in PK-15 cells. The PRV JM strain was isolated in our laboratory [10] and amplified in PK-15 cells.

### 2.2. Primers and Plasmid Construction

sgRNAs targeting the open reading frames of the PRV UL21/UL24/UL50/UL56 and gG genes were designed using the online CRISPR design tool (CRISPOR, http://crispor.tefor.net/). The plasmid pX330 was digested with BbsI enzyme (New England Biolabs, Beijing, China) to insert the sgRNA sequences. The sequences of the sgRNAs and primers used for mutant identification are listed in Table S1.

### 2.3. Recombinant Virus Construction Strategy

In this experiment, the construction of recombinant viruses was performed using the co-transfection method. A 6-well plate of 293T cells was prepared in advance, and when the cell density reached approximately 80%, transfection was carried out by co-transfecting the virus genomic DNA with pX330-sgRNA into the 293T cells. After 3–4 days, when the cells showed approximately 80% cytopathic effect (CPE), the cells were subjected to freeze–thaw cycles at −20 °C three times, and the virus supernatant was collected. The virus was diluted tenfold, and 2–4-fold diluted virus was inoculated onto PK-15 cells in a 6-well plate. After 2 h of incubation at 37 °C in a 5% CO_2_ incubator, the supernatant was discarded, and 1% FBS, 2% agarose-containing EMEM medium was added. After the medium solidified, the plate was inverted and cultured for 36 h. Once visible plaques appeared, individual plaques were picked and propagated in PK-15 cells. Each recombinant virus was subjected to five consecutive rounds of plaque purification to minimize residual parental virus and obtain clonally purified virus populations. After the fifth round of plaque purification, the purified viruses were amplified in PK-15 cells. Viral genomic DNA was subsequently extracted using the TIANamp Virus DNA Kit (TIANGEN, Beijing, China), and the intended genomic alterations at the target loci were confirmed by PCR and Sanger sequencing. The extracted viral genomic DNA was amplified using PrimeSTAR^®^ HS DNA Polymerase (Takara Bio Inc., Shiga, Japan) with mutant-specific primers. PCR amplification was performed with an initial denaturation at 98 °C for 3 min, followed by 35 cycles of denaturation at 98 °C for 10 s, annealing at 55 °C for 5 s, and extension at 72 °C for 3 min, followed by a final extension at 72 °C for 10 min. The PCR products were subsequently analyzed to verify the intended genomic disruptions.

### 2.4. PRV Multi-Step Growth Kinetics and Plaque Size Measurement

To measure the virus growth kinetics, PK-15 cells cultured in 6-well plates were infected with different virus strains at a multiplicity of infection (MOI) of 0.1 at 37 °C for 2 h. After 2 h, the supernatant was discarded, and the cells were replenished with DMEM containing 2% FBS. At 6, 12, 24, 36, 48 and 60 h post-infection, cell supernatants were collected to measure the viral titer. Virus titers in the collected supernatants were determined in Vero cells using the median tissue culture infectious dose (TCID_50_) method and expressed as TCID_50_/mL. To determine the plaque size produced by virus-infected cells, PK-15 monolayer cells in a 6-well plate were infected with different virus strains, incubated at 37 °C for 2 h, and then overlaid with EMEM mixed with 1% FBS and 2% agarose. After the medium solidified, the plate was incubated at 37 °C in a 5% CO_2_ incubator for 72 h. The plaques were stained with 1% crystal violet for 12 h, and the plaque size was observed at room temperature. For each virus strain, six wells containing different virus dilutions were used for plaque formation. After crystal violet staining, 10 well-separated plaques were randomly selected from wells containing clearly distinguishable plaques for plaque-area measurement. No predetermined microscopic fields were used for plaque selection.

### 2.5. Mice Experiment

A total of 35 female BALB/c mice, aged 4 weeks, were randomly divided into 7 groups, with 5 mice in each group. Mice were intramuscularly injected with 1 × 10^4^ TCID_50_ doses of the indicated PRV strain in a total volume of 100 μL. One group received 100 μL DMEM as a control. After injection, the mice were monitored for 14 days, observing their survival times, mortality, and clinical symptoms.

Mice that died during the 14-day observation period were necropsied promptly after death, and the brain, lung, liver, and spleen were collected for histopathological examination. Tissues from the DMEM control mice were collected at the end of the 14-day observation period.

### 2.6. Histopathological Analysis

The tissues were fixed for 24 h using a 10% aqueous formalin solution, after which they were embedded in paraffin wax. Sections of 3 μm thickness were cut, stained with hematoxylin and eosin (H&E), and then observed using a light microscope. Histopathological examination was performed descriptively; no predefined blinded semi-quantitative lesion-scoring system was included in the original experimental design.

### 2.7. Statistical Analysis

Statistical analyses were performed using GraphPad Prism 9.0 (GraphPad Software, San Diego, CA, USA). Data are presented as mean ± SD. Growth-kinetics data were analyzed using two-way ANOVA followed by Dunnett’s multiple-comparisons test, with each gene-disrupted mutant compared with PRV JM at each indicated time point. Plaque-area measurements were analyzed using one-way ANOVA followed by Tukey’s multiple-comparisons test. For plaque-area analysis, statistical comparisons were based on the measured plaque areas from randomly selected plaques in the plaque assay. Survival curves were compared using the log-rank (Mantel–Cox) test. A *p* value < 0.05 was considered statistically significant. Exact adjusted *p* values are reported where applicable.

### 2.8. Animal Ethics Statement

All animal experiments were performed in accordance with the guidelines for the care and use of laboratory animals and were approved by the Institutional Animal Care and Use Committee of Harbin Veterinary Research Institute (Approval No. 230808-01-GR; approval date: 8 August 2023).

## 3. Results

### 3.1. Construction of Five PRV JM Gene-Disrupted Mutants

**Figure 1:**
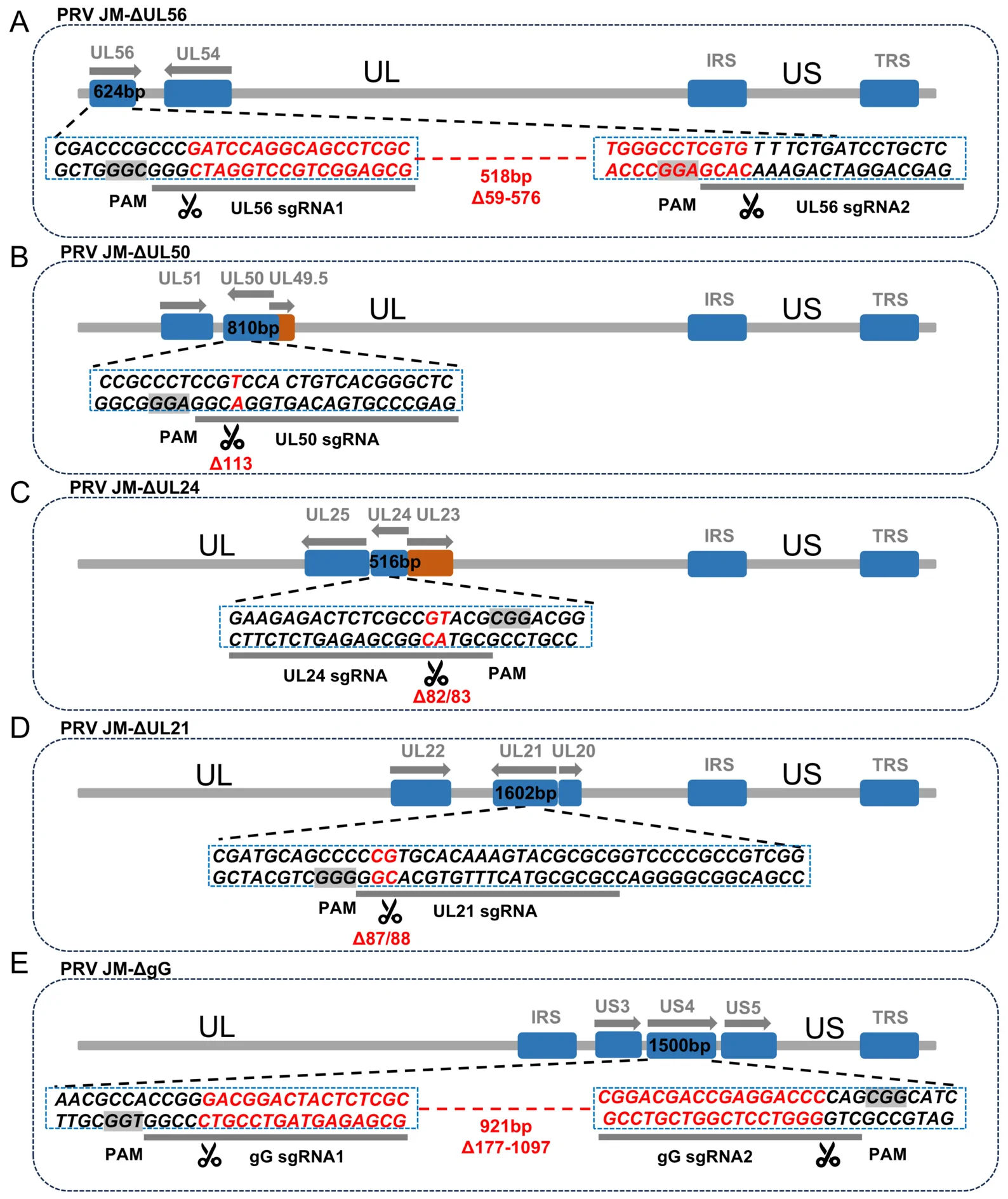
Schematic diagrams of CRISPR/Cas9-mediated construction of PRV gene-disrupted mutants. (**A**–**E**) Schematic representation of the sgRNA target sites within the PRV UL56, UL50, UL24, UL21, and gG genes. Red text indicates the sgRNA target sequences, scissors indicate the predicted Cas9 cleavage sites, and PAM sequences are underlined. Dashed boxes indicate the enlarged regions showing the sgRNA target sequences and predicted deletion sites. Dual-sgRNA targeting was used for UL56 and gG, with expected deletions of approximately 518 bp and 921 bp, respectively. Single-sgRNA targeting of UL50, UL24, and UL21 generated small deletions at nucleotide positions 113, 82–83, and 87–88, respectively, resulting in frameshift mutations predicted to cause premature translation termination.

**Figure 2:**
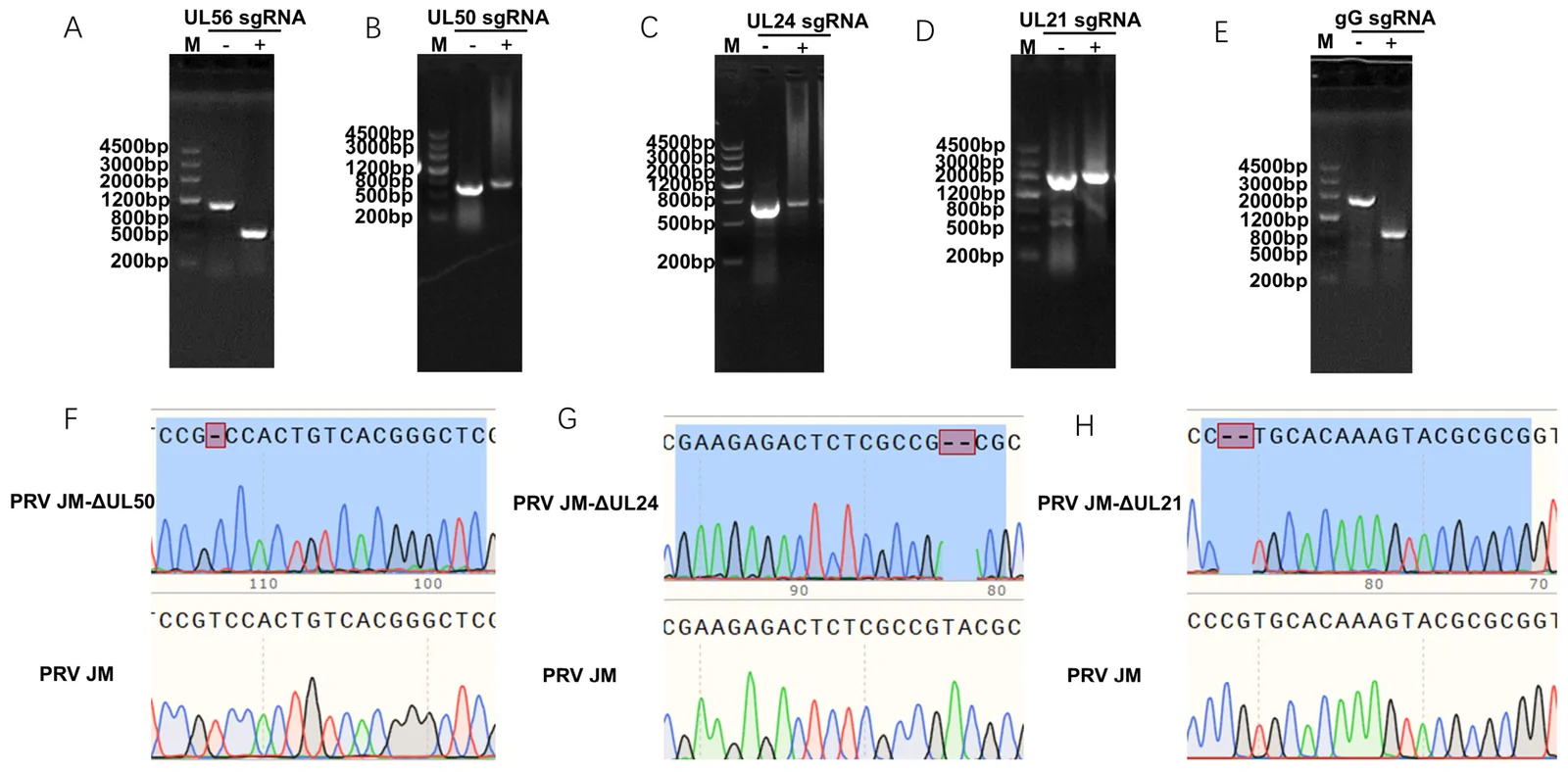
Construction and verification of PRV gene-disrupted mutants. (**A**–**E**) PCR analysis of the targeted loci in PRV JM and the corresponding gene-disrupted mutants; “−” indicates PRV JM and “+” indicates the corresponding recombinant mutant. Expected fragment reductions were observed for PRV JM-ΔUL56 (~518 bp) and PRV JM-ΔgG (~921 bp). (**F**–**H**) Representative Sanger sequencing chromatograms of the UL50, UL24, and UL21 target regions, respectively, showing the frameshift-inducing nucleotide deletions predicted to result in premature translation termination.Red boxes indicate the deleted nucleotide positions.

We selected the PRV UL21, UL24, UL50, UL56 and gG genes for targeted disruption in the PRV JM backbone, using the CRISPR/Cas9 system to construct gene-disrupted mutants, as shown in Figure 1. The pX330 plasmid carrying sgRNAs targeting each gene was co-transfected with the PRV JM genomic DNA into 293T cells. After cytopathic effects appeared, the virus supernatant was inoculated onto PK-15 cells for plaque formation. After plaque purification, PCR was performed using primers flanking each gene, and sequencing was conducted to confirm whether the mutants were successfully constructed. Because large deletions might affect the expression of adjacent genes, UL21, UL24, and UL50 were disrupted using a single sgRNA to induce frameshift mutations. Based on the sgRNA positions for each gene, PRV UL56 should have a 518 bp deletion, PRV gG a 921 bp deletion, and PRV UL50, PRV UL24, and PRV UL21 should exhibit frameshift mutations. The PCR results showed that after gene disruption, the fragment sizes for gG and UL56 were as expected, and sequencing confirmed that partial fragments were deleted. This indicates successful construction of PRV JM-ΔgG and PRV JM-ΔUL56 strains (Figure 2A–E). For PRV UL21, UL24, and UL50, only one sgRNA was used for disruption, so the confirmation was based on sequencing results. Sequencing revealed a deletion of nucleotides 87 and 88 in UL21, resulting in a frameshift mutation predicted to cause premature translation termination at amino acid 184. UL24 contained a deletion of nucleotides 82 and 83, resulting in a frameshift mutation predicted to cause premature translation termination at amino acid 40. UL50 contained a deletion of nucleotide 113, resulting in a frameshift mutation predicted to cause premature translation termination at amino acid 96 (Figure 2F–H).

### 3.2. Comparative Replication and Plaque Phenotypes of PRV Gene-Disrupted Mutants

**Figure 3:**
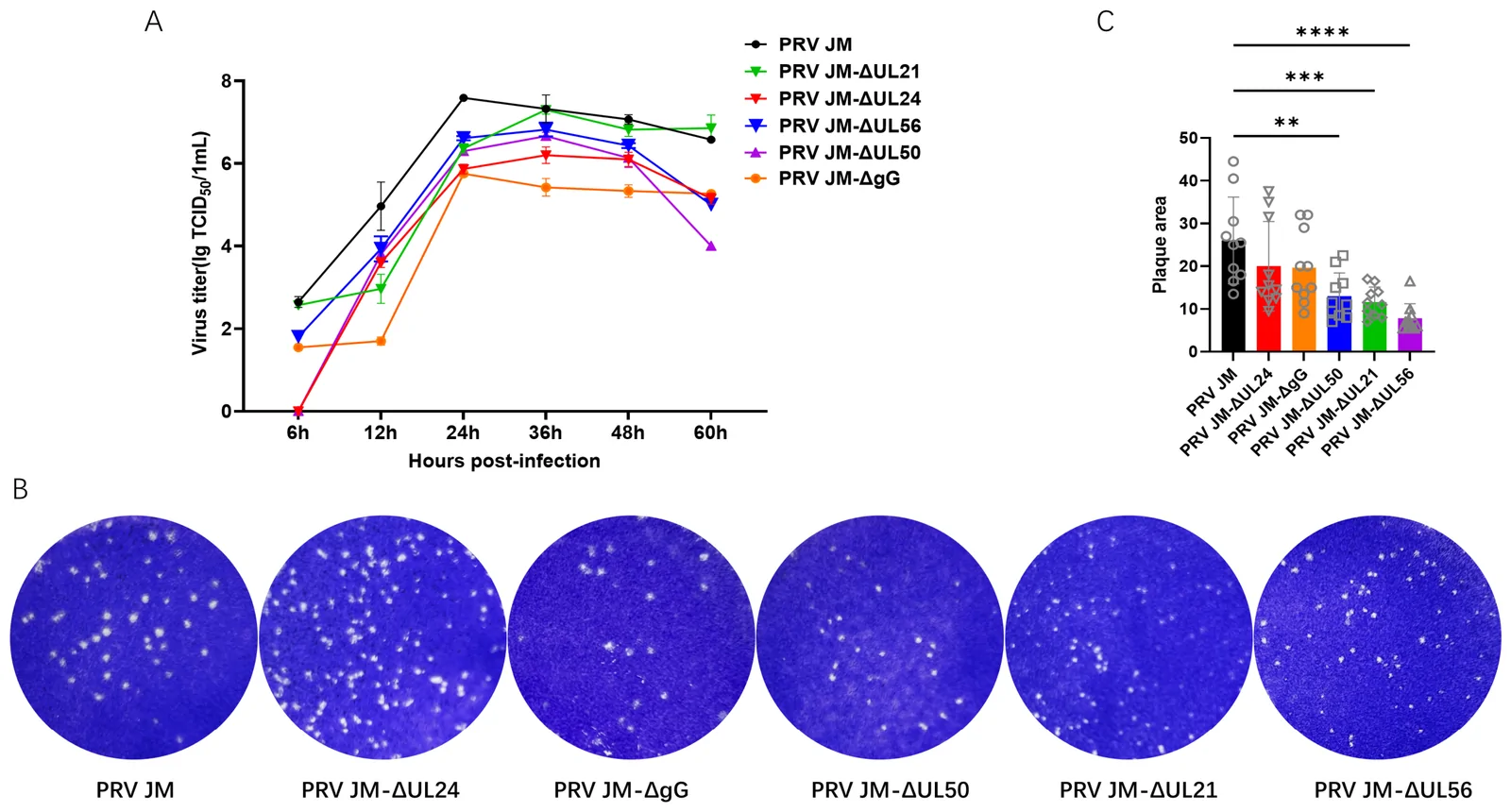
Growth kinetics and plaque morphology of PRV gene-disrupted mutants. (**A**) Multi-step growth curves of PRV JM and the indicated gene-disrupted mutants (MOI = 0.1) in PK-15 cells. Virus titers in the culture supernatants were determined in Vero cells using the TCID_50_ method. (**B**) Representative plaque morphologies of PRV JM and mutant strains at 72 h post-infection. (**C**) Quantification of plaque areas. For each virus strain, six wells containing different virus dilutions were used for plaque formation, and 10 well-separated plaques were randomly selected from wells in which individual plaques could be clearly distinguished. Data are presented as the mean ± SD of the 10 measured plaques. Statistical comparisons of plaque areas were performed using one-way ANOVA followed by Tukey’s multiple-comparisons test. The adjusted *p* values for comparisons with PRV JM were as follows: PRV JM vs. PRV JM-ΔUL24, *p* = 0.4544; PRV JM vs. PRV JM-ΔgG, *p* = 0.4001; PRV JM vs. PRV JM-ΔUL50, *p* = 0.0034; PRV JM vs. PRV JM-ΔUL21, *p* = 0.0010; and PRV JM vs. PRV JM-ΔUL56, *p* < 0.0001. ** *p* < 0.01, *** *p* < 0.001, and **** *p* < 0.0001.

To study the biological characteristics of the PRV gene-disrupted mutants, the PRV gene-disrupted mutants and the PRV JM strain were inoculated into PK-15 cells. Virus titers in the cell supernatants were measured at designated time points post-infection, and the multi-step growth curve was generated. From the multi-step growth curve, it was found that the PRV JM parental strain and PRV JM-ΔgG reached their peak viral titers at 24 h post-infection, whereas PRV JM-ΔUL21, PRV JM-ΔUL24, PRV JM-ΔUL50, and PRV JM-ΔUL56 reached their peak titers at 36 h post-infection. These data demonstrate distinct replication kinetics among the tested gene-disrupted viruses under the experimental conditions used. The peak viral titer of PRV JM was 7.60 log_10_ TCID_50_/mL. The corresponding peak titers of PRV JM-ΔUL21, PRV JM-ΔUL24, PRV JM-ΔUL50, PRV JM-ΔUL56, and PRV JM-ΔgG were 7.40, 6.25, 6.70, 6.60, and 5.80 log_10_ TCID_50_/mL, respectively, corresponding to reductions of 0.20, 1.35, 0.90, 1.00, and 1.80 log_10_ TCID_50_/mL relative to PRV JM. Overall, PRV JM-ΔUL24, PRV JM-ΔUL50, PRV JM-ΔUL56, and PRV JM-ΔgG showed reduced peak viral titers, whereas PRV JM-ΔUL21 showed a comparatively limited change in viral growth under the experimental conditions used (Figure 3A).

To further investigate the biological characteristics of PRV gene-disrupted mutants, the size of plaques was compared. The PRV gene-disrupted mutants and the wild-type PRV were inoculated onto a monolayer of PK-15 cells in 6-well plates. After incubation for 2 h, the virus solution was discarded and an EMEM mixture containing 1% fetal bovine serum and 2% agarose was added. After the mixture solidified, the culture plate was inverted and cultured for 72 h. Once obvious plaques appeared, crystal violet was added for staining, and the plaques were stained at room temperature for 12 h. Then, the size of the plaques was observed. The results showed that the plaque sizes of PRV JM-ΔUL24 and PRV JM-ΔgG mutants were not significantly different from those of the wild-type PRV, while the plaques of PRV JM-ΔUL50, PRV JM-ΔUL21, and PRV JM-ΔUL56 were significantly smaller than those of the wild-type PRV. Among them, PRV JM-ΔUL21 and PRV JM-ΔUL56 had the smallest plaques, a phenotype consistent with reduced local cell-to-cell spread under the plaque-assay conditions (Figure 3B). Ten well-separated plaques were randomly selected for each virus strain and their areas were quantified (Figure 3C).

### 3.3. Comparative Virulence Phenotypes of PRV Gene-Disrupted Mutants in Mice

**Figure 4:**
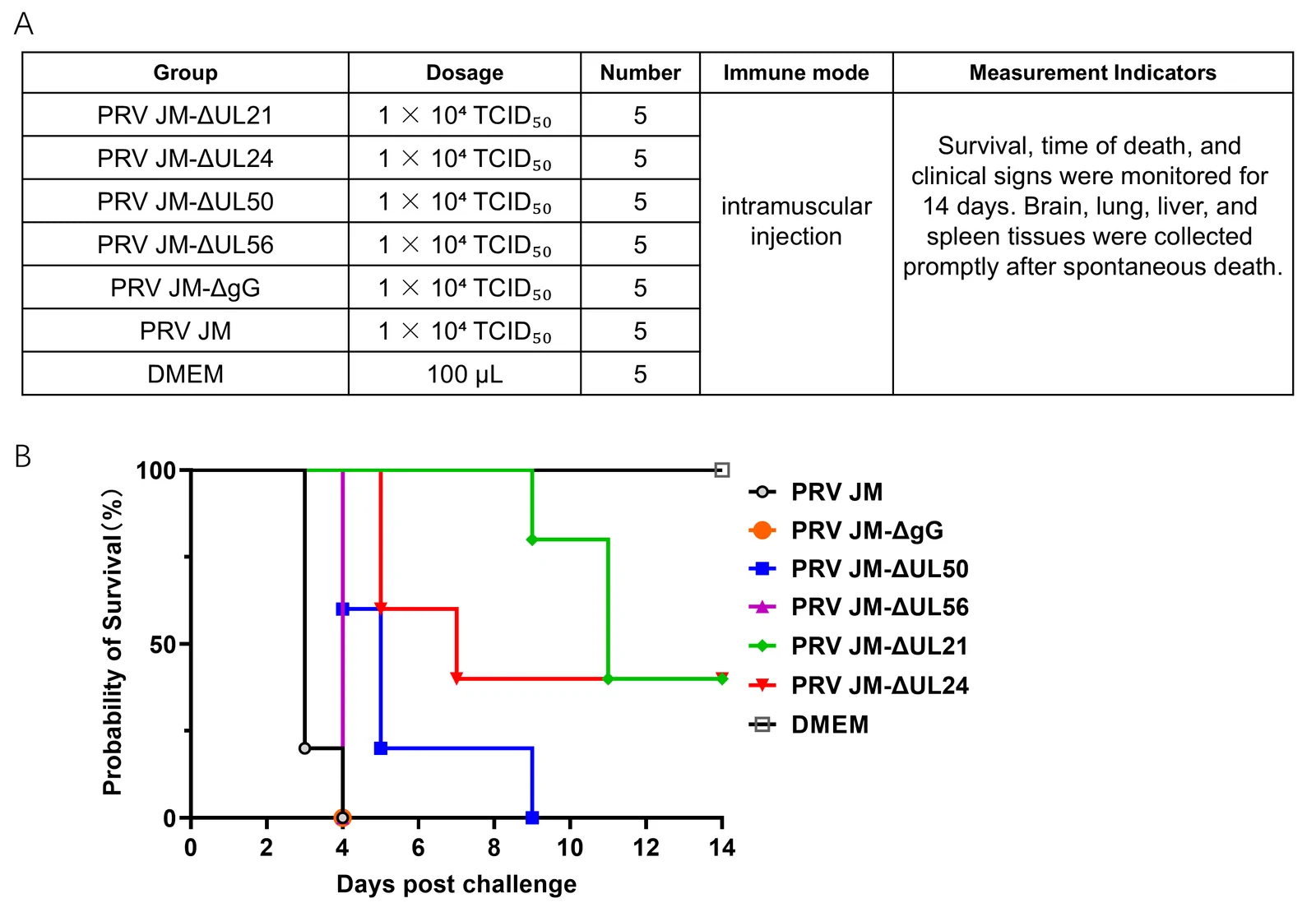
Comparative survival analysis of PRV gene-disrupted mutants in mice. (**A**) Experimental design of the animal infection assay. BALB/c mice (*n* = 5 per group) were inoculated intramuscularly with 1 × 10^4^ TCID_50_ of the indicated PRV strain in a total volume of 100 μL. The control group received 100 μL of DMEM via the same route. Mice were monitored for clinical signs and survival for 14 days. (**B**) Kaplan–Meier survival curves of mice in the PRV JM, PRV JM-ΔUL21, PRV JM-ΔUL24, PRV JM-ΔUL50, PRV JM-ΔUL56, PRV JM-ΔgG, and DMEM control groups. Survival curves were compared using the log-rank (Mantel–Cox) test.

To compare the virulence phenotypes associated with disruption of UL21, UL24, UL50, UL56, and gG, mice were divided into seven groups and inoculated intramuscularly with 1 × 10^4^ TCID_50_ of the indicated PRV strain in a total volume of 100 μL. The control group received 100 μL of DMEM via the same route. Mice in the PRV JM group began to die on day 3 and all died by day 4, whereas no deaths or clinical signs were observed in the DMEM control group. Mortality in the PRV JM-ΔUL21 group began on day 9, with 40% of the mice surviving to the end of the observation period. Mortality in the PRV JM-ΔUL24 group began on day 5, with a final survival rate of 40%. Mice in the PRV JM-ΔUL50 group began to die on day 5 and all died by day 9. All mice in the PRV JM-ΔUL56 and PRV JM-ΔgG groups died by day 4 (Figure 4). Overall, disruption of UL21 or UL24 was associated with comparatively greater attenuation in the mouse model, whereas PRV JM-ΔUL56 and PRV JM-ΔgG showed survival patterns more similar to that of the parental PRV JM strain.

### 3.4. Histopathological Observations in Mice Infected with PRV Gene-Disrupted Mutants

**Figure 5:**
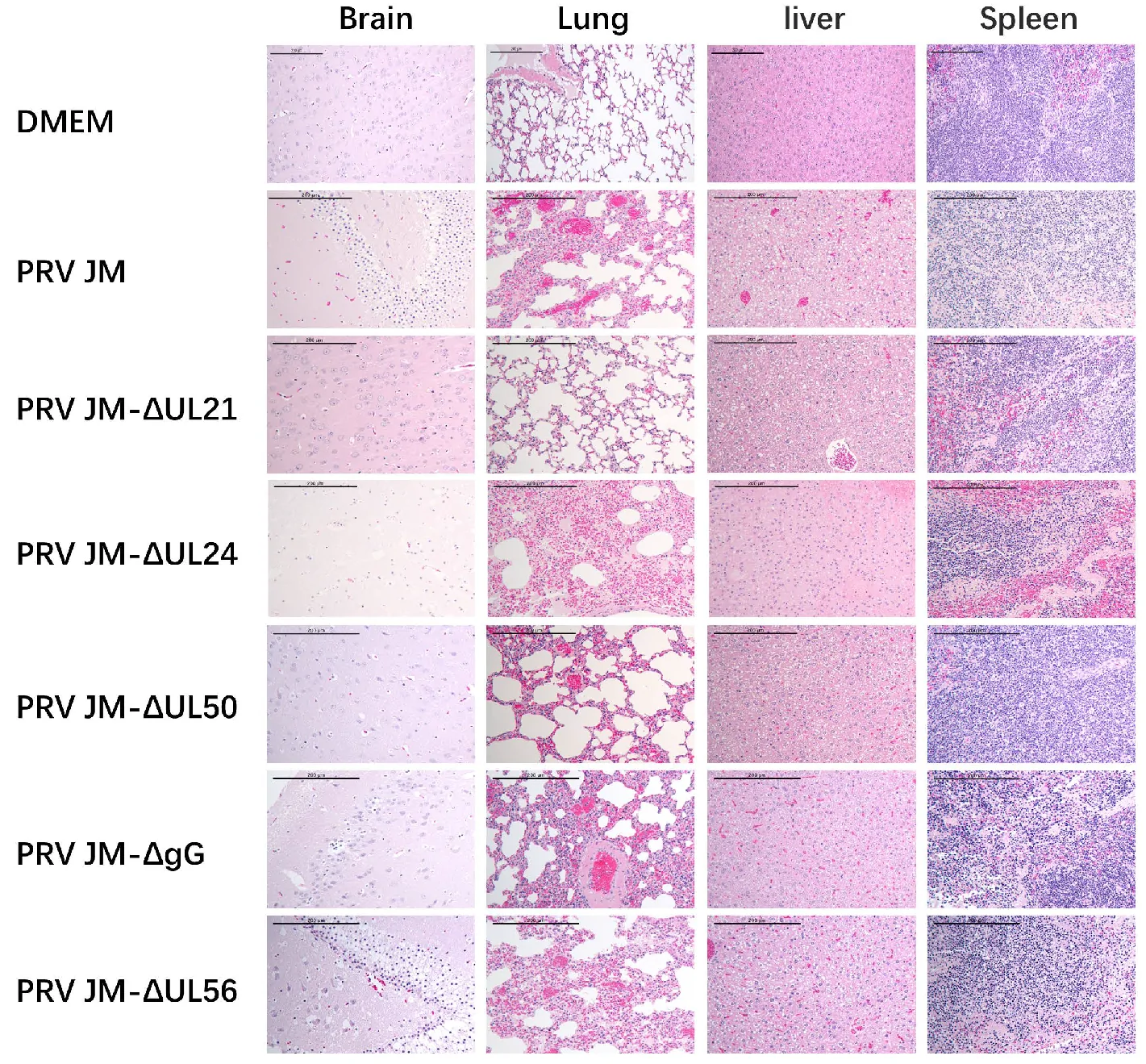
Histopathological examination of tissues from control and PRV-infected mice. Representative hematoxylin and eosin (H&E)-stained sections of the brain, lung, liver, and spleen from the DMEM control group and mice infected with PRV JM, PRV JM-ΔUL21, PRV JM-ΔUL24, PRV JM-ΔUL50, PRV JM-ΔUL56, or PRV JM-ΔgG are shown. Tissues from infected mice were collected promptly after spontaneous death during the 14-day observation period, whereas control tissues were collected at the end of the observation period. Scale bars = 200 μm. These sections are presented for descriptive morphological assessment and were not subjected to blinded semi-quantitative pathological scoring.

Mice that died during the 14-day observation period were necropsied promptly after death, and the brain, lung, liver, and spleen were collected for histopathological examination. In the PRV JM group, the brain showed degeneration of hippocampal neurons accompanied by nuclear condensation. The lungs exhibited congestion of capillaries and small veins in the alveolar walls, mild thickening of the alveolar walls, and hyperplasia of alveolar epithelial cells. The liver showed hepatocellular degeneration. The spleen exhibited poorly defined lymphoid follicles and reduced lymphocyte numbers. Mice infected with PRV JM-ΔUL24 showed no obvious histopathological changes in the brain. The lungs exhibited congestion of small veins and capillaries in the alveolar walls, accompanied by hemorrhage and serous exudation in the alveolar lumen. Hepatocellular degeneration was observed in the liver. In the spleen, an increased accumulation of red blood cells and reduced lymphocyte numbers in the white pulp were observed. Mice infected with PRV JM-ΔUL21 showed no obvious histopathological changes in the brain, lungs, or spleen. Hepatocellular degeneration was observed in the liver. Mice infected with PRV JM-ΔUL50 showed no obvious histopathological changes in the brain or lungs. Hepatocellular degeneration was observed in the liver, and focal lymphocyte necrosis was observed in the splenic white pulp. Mice infected with PRV JM-ΔUL56 showed swelling, degeneration, and nuclear condensation of hippocampal neurons. The lungs exhibited congestion of capillaries and small veins in the alveolar walls, accompanied by serous exudation in the alveolar lumen and interstitium. Hepatocellular degeneration was observed in the liver. In the spleen, lymphocyte numbers in the white pulp were reduced, with focal lymphocyte necrosis. Mice infected with PRV JM-ΔgG exhibited glial-cell infiltration in the brain. The lungs showed thickening of the alveolar walls, capillary congestion, and inflammatory-cell infiltration. Hepatocellular degeneration was observed in the liver, whereas lymphocyte numbers in the spleen were reduced. Representative H&E sections revealed heterogeneous histopathological alterations in the examined tissues among the infected groups (Figure 5). Because the histopathological component of the original experiment was designed for descriptive morphological assessment rather than predefined blinded semi-quantitative scoring, these observations are presented descriptively and are not intended to provide quantitative comparisons of lesion severity among groups. Accordingly, the survival analysis represents the principal quantitative in vivo comparison in the present study, whereas the histopathological findings provide supportive morphological context.

## 4. Discussion

In this study, CRISPR/Cas9 technology was used to construct single-gene-disrupted mutants of the UL21, UL24, UL50, UL56, and gG immune regulatory genes in the background of the PRV JM strain. Through comparative analyses of viral growth kinetics, plaque formation, mouse mortality, and tissue pathology, we characterized the distinct phenotypes observed among these five gene-disrupted viruses. This side-by-side comparison provides information on the comparative phenotypic differences associated with disruption of these loci and may help prioritize candidates for subsequent mechanistic studies.

An interesting finding was the dissociation between viral yield and plaque phenotype. PRV JM-ΔUL24 and PRV JM-ΔgG showed substantially reduced infectious titers in culture supernatants, but their plaque sizes were not significantly different from those of PRV JM. Conversely, PRV JM-ΔUL21 showed relatively little change in viral growth but exhibited markedly smaller plaques. These observations suggest that reductions in extracellular infectious virus production do not necessarily reflect impaired local cell-to-cell spread. Viral titers in culture supernatants are influenced by multiple stages of the replication cycle, including genome replication, virion assembly, maturation, release, and the infectivity of progeny particles, whereas plaque formation primarily reflects the ability of infection to propagate locally under semi-solid overlay conditions. Therefore, the reduced titers observed for PRV JM-ΔUL24 and PRV JM-ΔgG may involve stages other than local cell-to-cell spread. However, because these individual steps were not directly examined, the underlying mechanisms require further investigation.

Among the five mutants, PRV JM-ΔUL21 and PRV JM-ΔUL24 showed comparatively greater attenuation in mice, although both viruses still caused 60% mortality. Previous studies support the involvement of UL21 in PRV neuroinvasion and virulence. Mutations in UL21 have been associated with the attenuated phenotype of the Bartha vaccine strain, and repair of the UL21 locus enhanced retrograde and transneuronal infection [6,11]. In addition, the C-terminal region of PRV pUL21 contributes to neuroinvasion [12]. These previous observations are consistent with the delayed mortality observed for PRV JM-ΔUL21 in the present study. Similarly, previous disruption of PRV UL24 was reported to reduce virulence in mice [5], and UL24 proteins have been implicated in herpesvirus immune evasion [13]. Thus, the comparatively attenuated phenotype of PRV JM-ΔUL24 is consistent with previous observations. Nevertheless, the substantial residual mortality of both mutants demonstrates that neither PRV JM-ΔUL21 nor PRV JM-ΔUL24 exhibited an attenuation phenotype sufficient to support consideration as a safely attenuated strain under the present experimental conditions. Rather, these genes may represent candidate loci for further evaluation in combination with other attenuation-associated modifications.

In contrast, the UL50-, UL56-, and gG-disrupted viruses exhibited different combinations of in vitro and in vivo phenotypes. PRV JM-ΔUL50 showed reduced viral replication and delayed mortality but ultimately caused 100% mortality under the conditions used here. Previous studies have identified PRV UL50 as a viral dUTPase involved in antagonizing type I interferon signaling [7,14,15,16], and dUTPase-deficient PRV has been reported to be attenuated in pigs [14]. PRV JM-ΔUL56, despite exhibiting reduced viral titers and smaller plaques, showed a mortality pattern similar to that of PRV JM. This is notable because UL56 has previously been implicated in MHC-I downregulation and PRV dissemination and virulence [8,17,18]. These differences may reflect strain background, host species, or experimental conditions. PRV JM-ΔgG showed the largest reduction in peak viral titer among the five mutants but exhibited a survival pattern similar to that of parental PRV JM, further emphasizing that impaired replication in cultured cells does not necessarily predict attenuation in vivo. Together, these results highlight the importance of evaluating candidate attenuation loci using both in vitro and in vivo phenotypes rather than relying on viral growth characteristics alone.

Histopathological examination provided descriptive morphological information accompanying the survival analysis. Representative sections revealed neuronal, pulmonary, hepatic, and splenic alterations in several infected groups. However, because the histopathological component of the original study was not designed using a predefined blinded semi-quantitative scoring system, these observations should not be interpreted as quantitative evidence of differences in lesion severity among groups. Accordingly, survival represents the principal quantitative in vivo endpoint in the present study, whereas the histopathological findings provide supportive morphological context.

The pathological findings should also be interpreted in relation to the experimental host. Pigs are the natural host of PRV, whereas mice represent a highly susceptible non-natural host used here primarily for comparative assessment of virulence phenotypes. In pigs, PRV pathogenesis is strongly influenced by host age and viral virulence. Experimental studies have shown substantially more pronounced viral replication and neurological disease in young piglets than in older pigs, together with age-dependent differences in viral replication within the trigeminal ganglia and central nervous system [19]. In addition, intranasal infection studies in pigs have demonstrated strain-dependent differences in nasal mucosal damage, neural invasion, and the kinetics of viral spread [20]. Pulmonary and neurological lesions have also been documented during infection with virulent PRV strains in pigs [21,22], and recent experimental work has further demonstrated PRV-associated inflammatory lung injury in infected piglets [23]. Therefore, although neuronal degeneration, pulmonary congestion, and alveolar alterations observed in the present mouse model share some qualitative features with pathological changes reported in pigs, the distribution, severity, and clinical significance of these lesions cannot be directly extrapolated to natural porcine infection. The murine model used here should thus primarily be regarded as a standardized comparative system for evaluating relative survival phenotypes among the tested viruses. Confirmation in pigs will be required to determine whether the relative phenotypic patterns observed in mice are maintained in the natural host.

Several limitations should be considered when interpreting these findings. First, although the intended genomic alterations were confirmed by PCR and Sanger sequencing, protein-level validation was not performed for UL21-, UL24-, and UL50-disrupted viruses; therefore, complete absence of the corresponding full-length proteins cannot be directly demonstrated. Second, revertant or genetically complemented viruses were not generated, and whole-genome sequencing was not performed. Consequently, the observed phenotypes should be interpreted as being associated with the targeted genomic disruptions rather than as definitive evidence of direct gene-specific causality. Potential effects of the introduced mutations on neighboring genes or local cis-regulatory elements also cannot be completely excluded. Third, although each recombinant virus underwent five consecutive rounds of plaque purification, long-term genetic stability during serial passage was not systematically evaluated. In addition, the plaque-size measurements provide supportive evidence of altered plaque morphology but require confirmation in independently replicated experiments. Finally, histopathological analysis was descriptive, and the animal experiments included only five mice per group. Further studies incorporating genetic rescue, genome-wide sequence validation, independent phenotypic replication, and evaluation in pigs will therefore be required.

## 5. Conclusions

In summary, side-by-side characterization of five gene-disrupted viruses in the same PRV JM background revealed that changes in viral replication, plaque formation, and virulence were not necessarily concordant. Reduced extracellular viral yield did not consistently correspond to reduced plaque size or attenuation in mice, underscoring the importance of evaluating candidate attenuation loci across multiple phenotypic levels. Among the viruses examined, PRV JM-ΔUL21 and PRV JM-ΔUL24 displayed the most pronounced attenuation-associated phenotypes in mice, whereas PRV JM-ΔUL50 showed a more limited delay in mortality and PRV JM-ΔUL56 and PRV JM-ΔgG retained substantial pathogenicity despite measurable alterations in vitro. These findings prioritize UL21 and UL24 for further investigation as components of multi-gene attenuation strategies and provide a comparative framework for evaluating additional PRV attenuation-associated loci. Further genetic and protein-level validation, together with studies in the natural porcine host, will be necessary to define the gene-specific mechanisms underlying these phenotypes and their relevance to vaccine-oriented attenuation strategies.

## Data Availability

The original contributions presented in this study are included in the article/Supplementary Material. Further inquiries can be directed to the corresponding author.

## Supplementary Materials

The following supporting information can be downloaded at: https://www.mdpi.com/article/10.3390/vetsci13090867/s1, File S1 Original image of Figure F2 ABCDE F3B, Table S1: Sequences of the sgRNAs and primers used for mutant identification.
