# Supplementary material for: Comparative Phenotypic Characterization of Five CRISPR/Cas9-Generated Gene-Disrupted Mutants of Pseudorabies Virus JM Strain
Source: Vet Sci. 2026 Aug 26;13(9):867. doi: 10.3390/vetsci13090867 (PMC13612093; doi:10.3390/vetsci13090867)
Supplement: Supplementary file 1 [file vetsci-13-00867-s001.zip › vetsci-4463924-original-images.pdf]

Figure2A

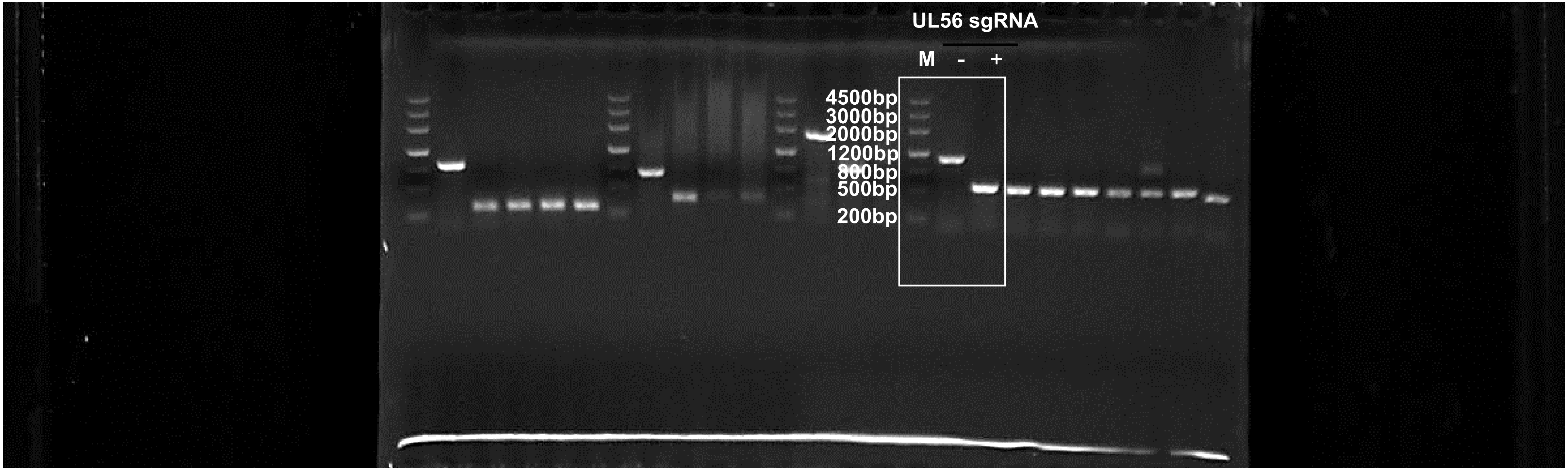

Figure 2. Construction and verification of PRV gene-deletion mutants.
(A–E) PCR analysis of PRV UL56, UL50, UL24, UL21, and gG deletions; "–" indicates wild-type PRV JM, "+" indicates recombinant mutants. Expected fragment reductions were observed for PRVΔUL56 (~680 bp) and PRVΔgG (~640 bp).

Figure2B

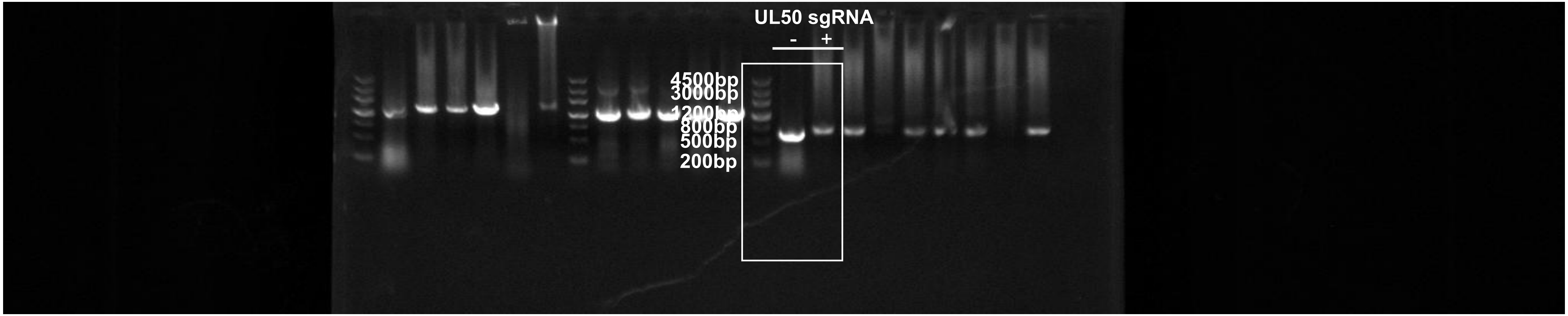

Figure 2. Construction and verification of PRV gene-deletion mutants.
(A–E) PCR analysis of PRV UL56, UL50, UL24, UL21, and gG deletions; "–" indicates wild-type PRV JM, "+" indicates recombinant mutants. Expected fragment reductions were observed for PRVΔUL56 (~680 bp) and PRVΔgG (~640 bp).

Figure2C

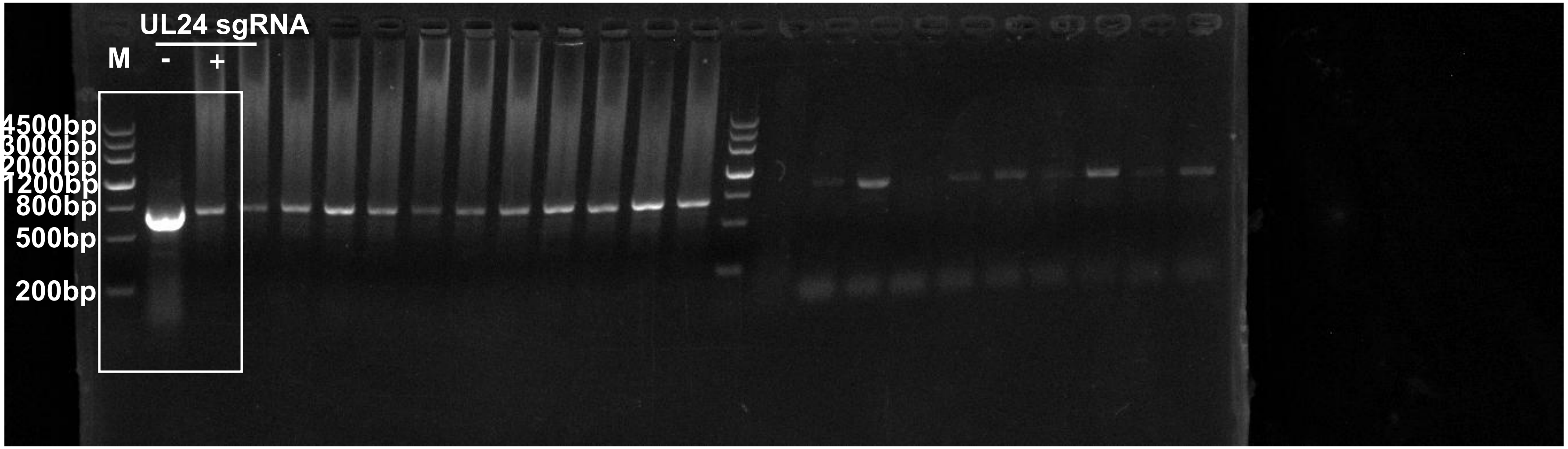

Figure 2. Construction and verification of PRV gene-deletion mutants.
(A–E) PCR analysis of PRV UL56, UL50, UL24, UL21, and gG deletions; "–" indicates wild-type PRV JM, "+" indicates recombinant mutants. Expected fragment reductions were observed for PRVΔUL56 (~680 bp) and PRVΔgG (~640 bp).

Figure2D

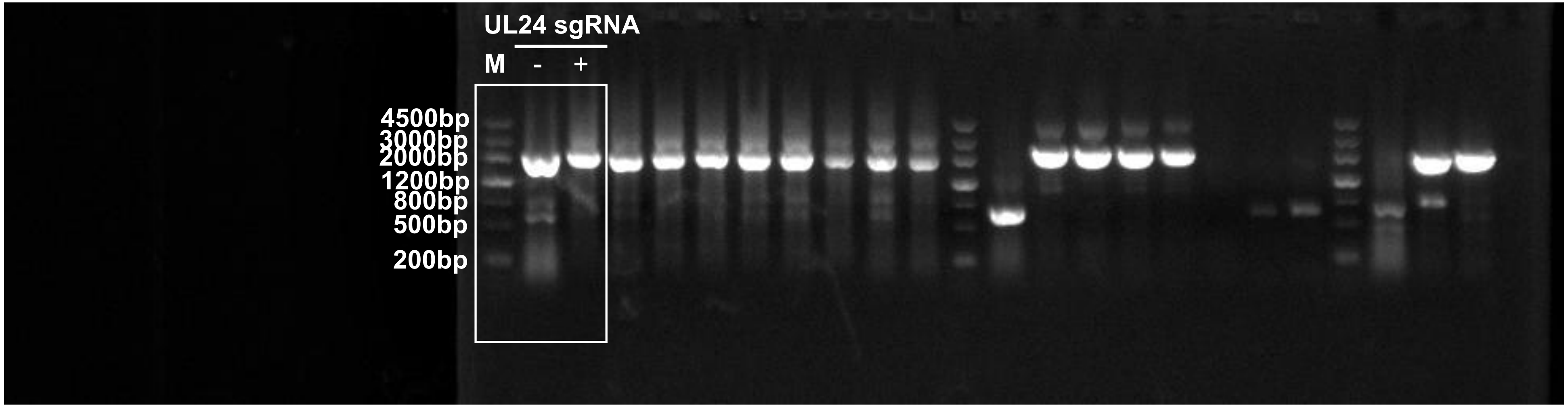

Figure 2. Construction and verification of PRV gene-deletion mutants.
(A–E) PCR analysis of PRV UL56, UL50, UL24, UL21, and gG deletions; "–" indicates wild-type PRV JM, "+" indicates recombinant mutants. Expected fragment reductions were observed for PRVΔUL56 (~680 bp) and PRVΔgG (~640 bp).

Figure2E

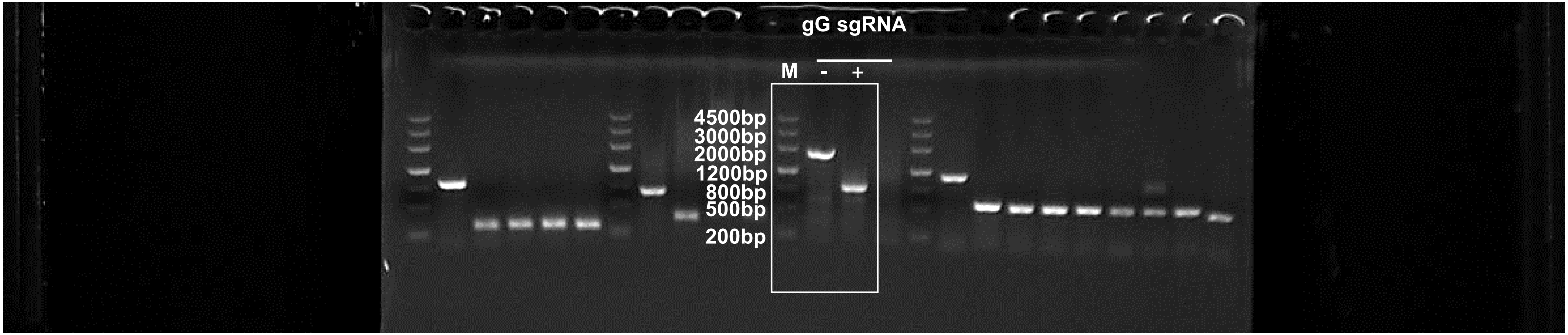

Figure 2. Construction and verification of PRV gene-deletion mutants.
(A–E) PCR analysis of PRV UL56, UL50, UL24, UL21, and gG deletions; "–" indicates wild-type PRV JM, "+" indicates recombinant mutants. Expected fragment reductions were observed for PRVΔUL56 (~680 bp) and PRVΔgG (~640 bp).

Figure 3B

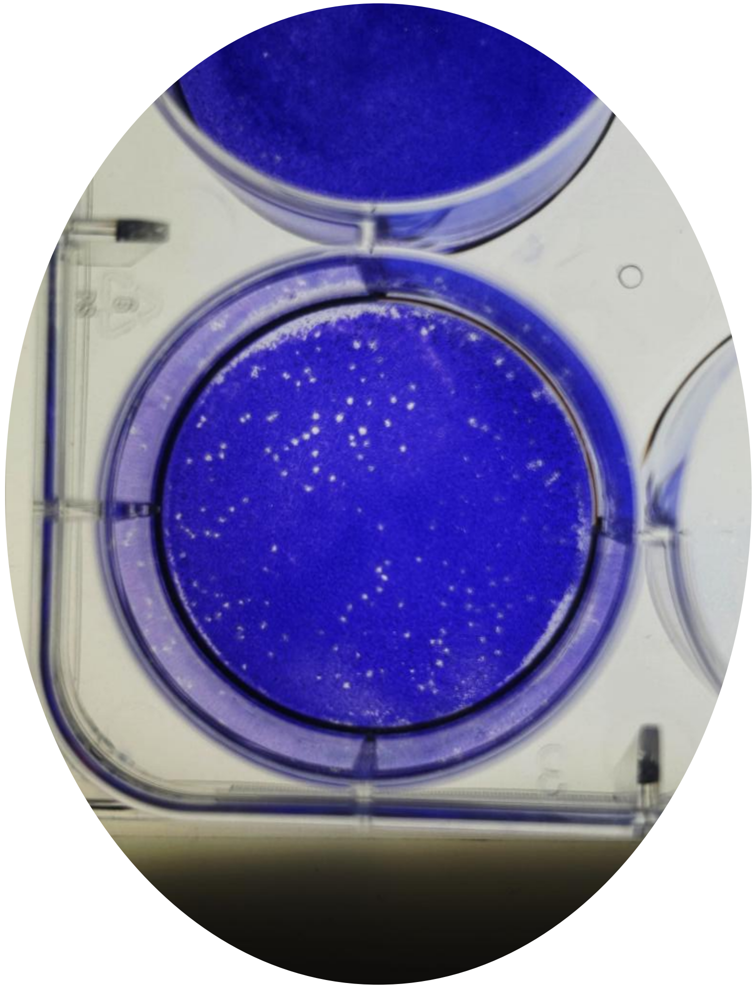

PRV JM

Figure 3B

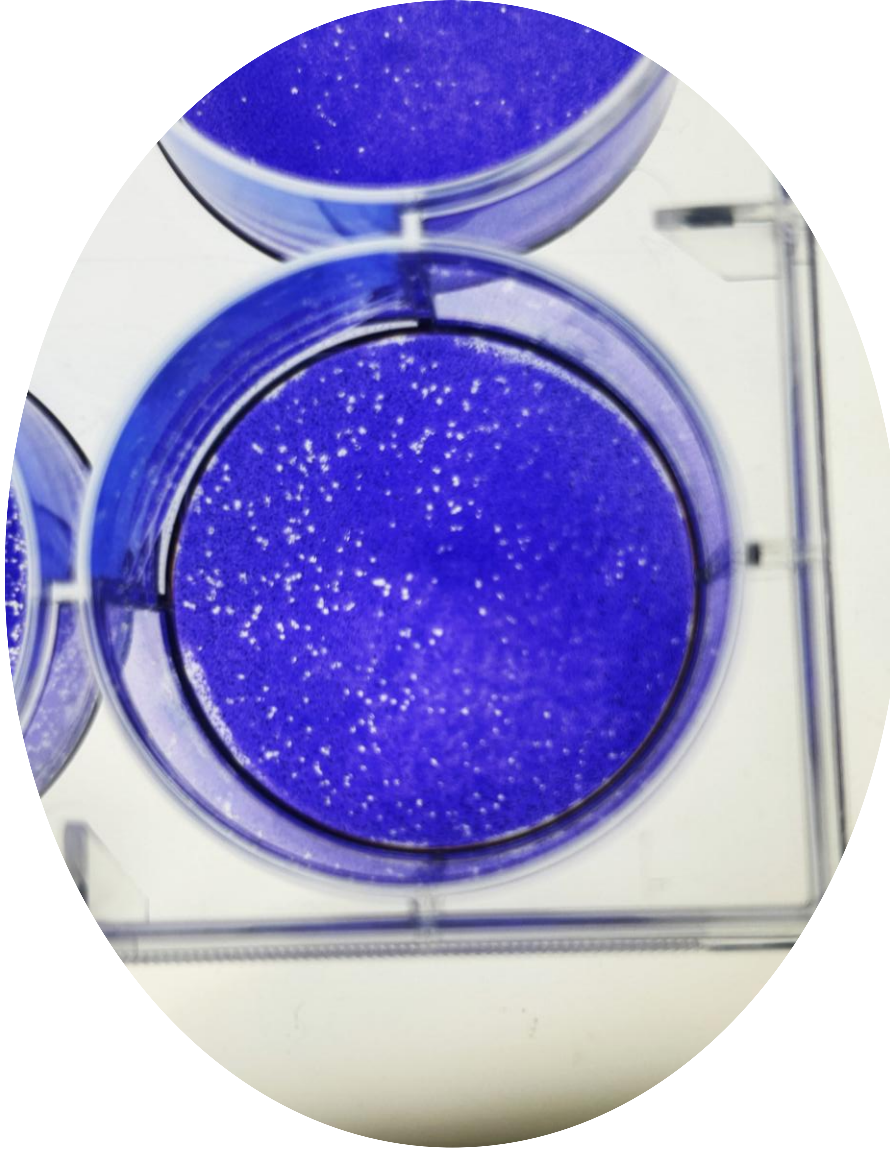

PRV JM-ΔUL24

Figure 3B

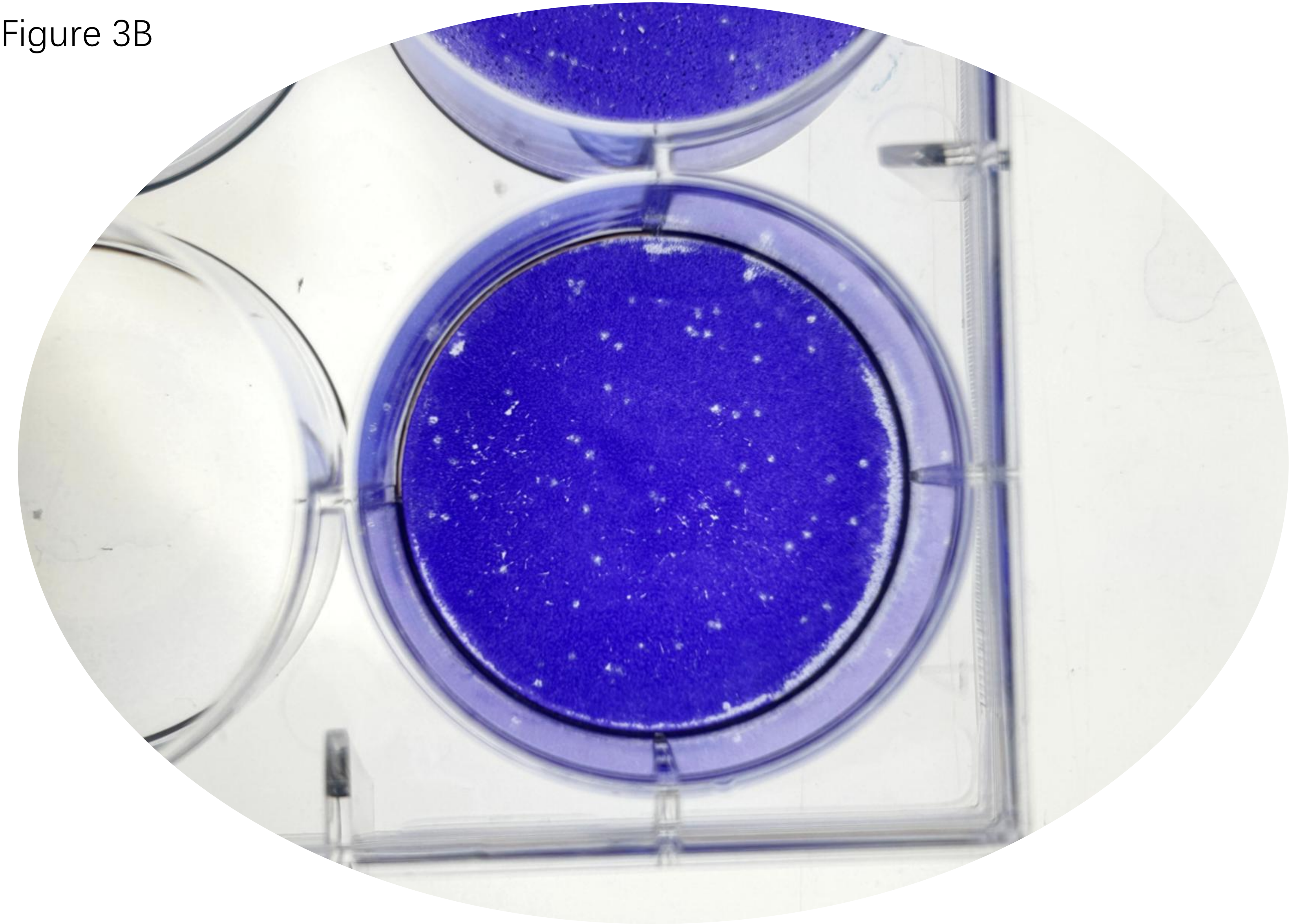

PRV JM-ΔUS4

Figure 3B

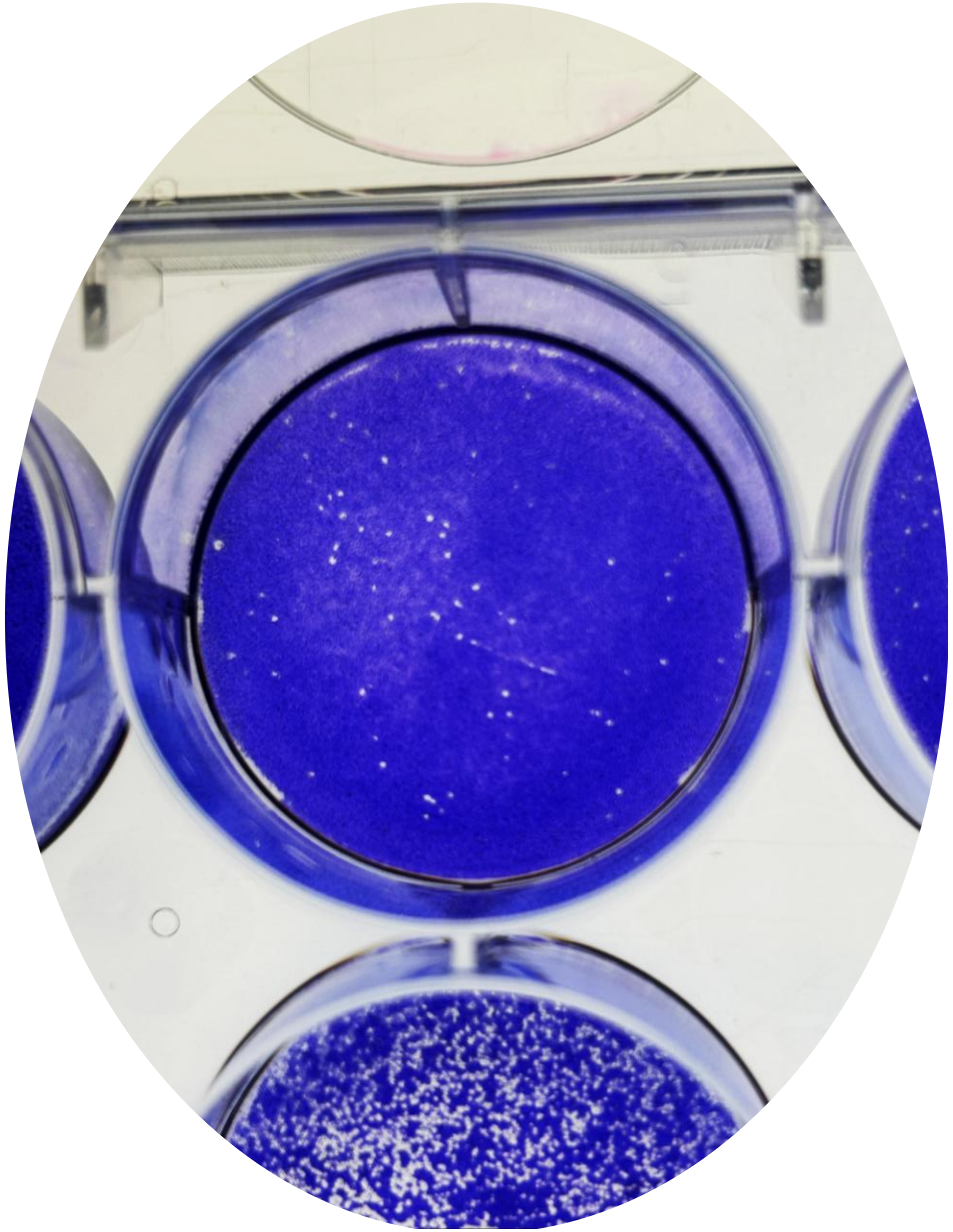

PRV JM-ΔUL50

Figure 3B

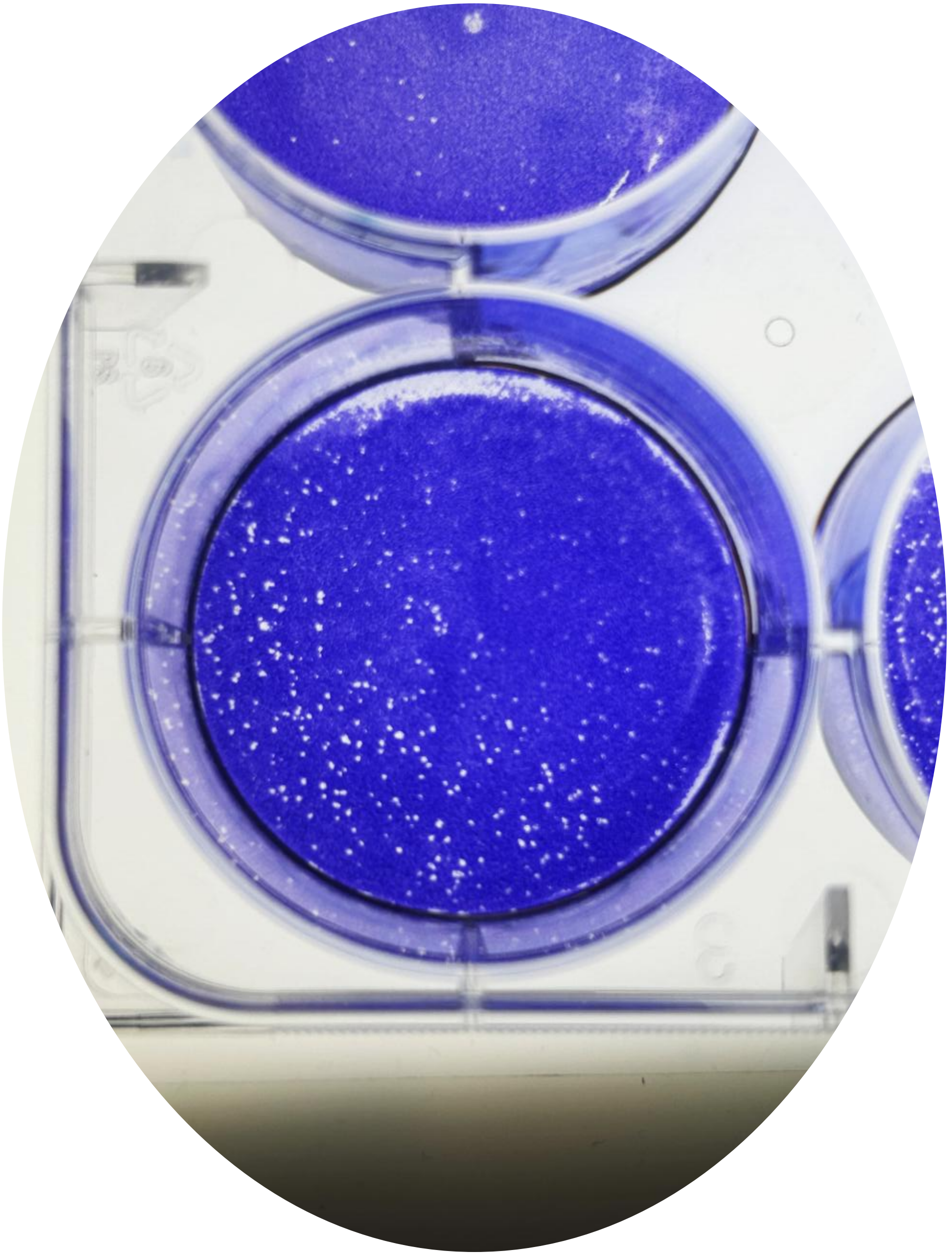

PRV JM-ΔUL21

Figure 3B

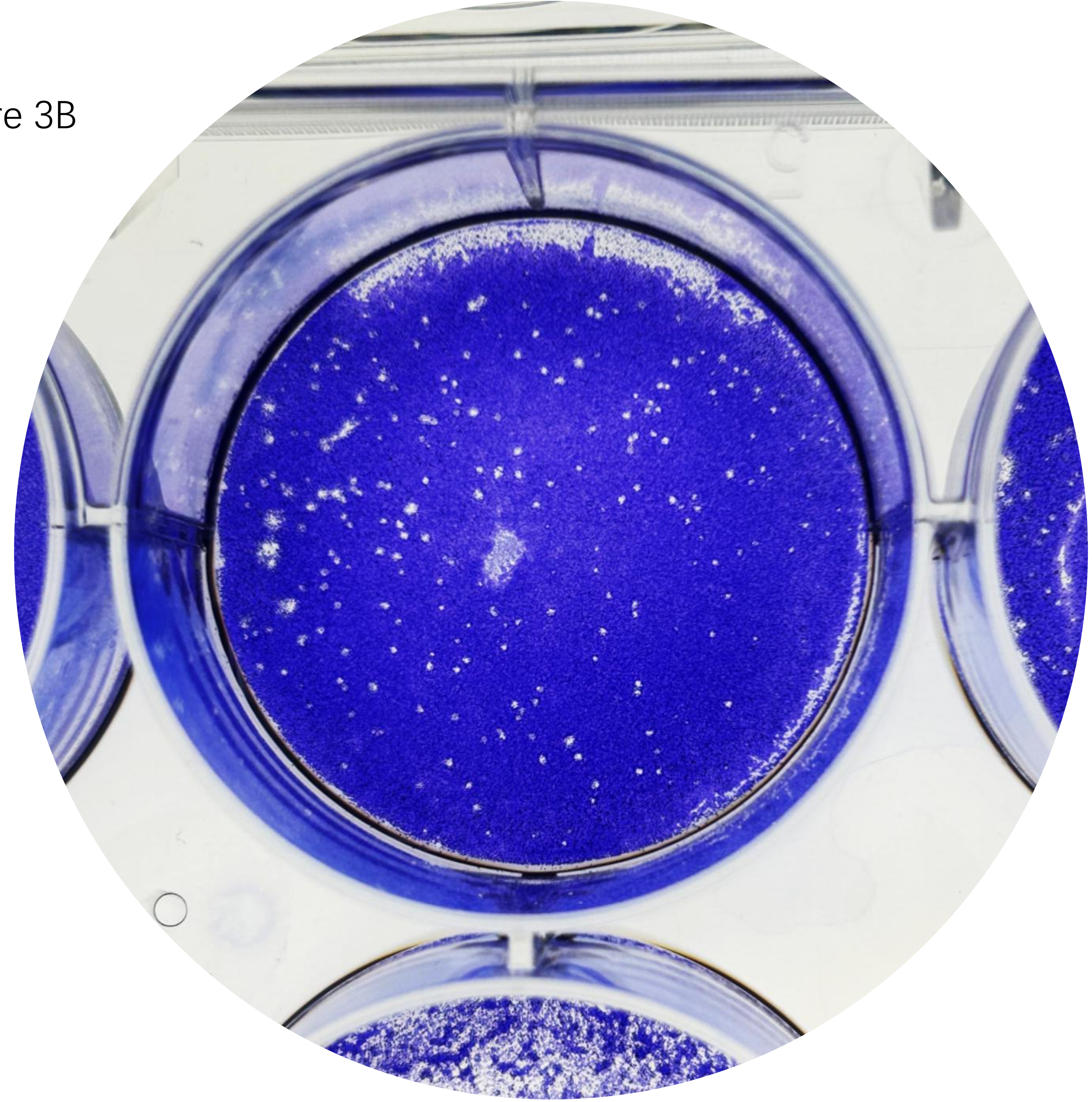

PRV JM-ΔUL56
