# Supplementary material for: Comparative Phenotypic Characterization of Five CRISPR/Cas9-Generated Gene-Disrupted Mutants of Pseudorabies Virus JM Strain
Source: Vet Sci. 2026 Aug 26;13(9):867. doi: 10.3390/vetsci13090867 (PMC13612093; doi:10.3390/vetsci13090867)
Supplement: Supplementary file 1 [file vetsci-13-00867-s001.zip › vetsci-4463924-supplementary.pdf]

Table S1 The sequences of the sgRNAs and primers used for mutant identification.

UL24sgRNA-F	CACCGAAGAGACTCTCGCCGTACG
UL24sgRNA-R	AAACCGTACGGCGAGAGTCTCTTC
UL24 identify-F	AACGGGGGCGCGATGAAGTGGC
UL24 identify-R	TAGGCGCCGTCGAGGTAGATC
UL50sgRNA-F	CACCGAGCCCGTGACAGTGGACGG
UL50sgRNA-R	AAACCCGTCCACTGTCACGGGCTC
UL50 identify-F	TCGGAAAAGACTATGGAAGAG
UL50 identify-R	AGAAGCGTCCTGCGTGTGTTG
UL21sgRNA-F	CACCCGCGCGTACTTTGTGCACGG
UL21sgRNA-R	AAACCCGTGCACAAAGTACGCGCG
UL21 identify-F	TGTGTGTGCCGCGGACAGAGAC
UL21 identify-R	CGCCTCGCTCACCCATTTGC
US4sgRNA1-F	CACCGCGAGAGTAGTCCGTCCCGG
US4sgRNA1-R	AAACCCGGGACGGACTACTCTCGC
US4sgRNA2-F	CACCCGGACGACCGAGGACCCCAG
US4sgRNA2-R	AAACCTGGGGTCCTCGGTCGTCCG
US4 identify-R	ACCGCTGGGCGTGGATCGCAC
US4 identify-F	TCCTGATTTACAAGATGTTGACG
UL56sgRNA1-F	CACCGCGAGGCTGCCTGGATCGGG
UL56sgRNA1-R	AAACCCCGATCCAGGCAGCCTCGC
UL56sgRNA2-F	CACCGGAGCAGGATCAGAAACACG
UL56sgRNA2-R	AAACCGTGTTTCTGATCCTGCTCC
UL56 identify-F	TGAGGCCGGAGAGAGACAGGGAGAGATG
UL56 identify-R	GCTTCACCGTCCCTTCCGCACACTC
